# A Novel, Highly Potent NADPH-Dependent Cytochrome P450 Reductase from Waste *Liza klunzingeri* Liver

**DOI:** 10.3390/md21020099

**Published:** 2023-01-29

**Authors:** Soudeh Bahramian Nasab, Ahmad Homaei, Roberto Fernandez-Lafuente, Jon Del Arco, Jesús Fernández-Lucas

**Affiliations:** 1 Department of Marine Biology, Faculty of Marine Science and Technology, University of Hormozgan, Bandar Abbas P.O. Box 3995, Iran; 2 Departamento de Biocatálisis, ICP-CSIC, Campus UAM-CSIC, 28049 Madrid, Spain; 3 Applied Biotechnology Group, Universidad Europea de Madrid Urbanización El Bosque, E-28670 Villaviciosa de Odón, 28670 Madrid, Spain; 4 Grupo de Investigación en Ciencias Naturales y Exactas, GICNEX, Universidad de la Costa, CUC, Calle 58 # 55-66, Barranquilla 080002, Colombia

**Keywords:** marine enzymes, cytochrome P450 reductase, purification, biotechnology

## Abstract

The use of marine enzymes as catalysts for biotechnological applications is a topical subject. Marine enzymes usually display better operational properties than their animal, plant or bacterial counterparts, enlarging the range of possible biotechnological applications. Due to the fact that cytochrome P450 enzymes can degrade many different toxic environmental compounds, these enzymes have emerged as valuable tools in bioremediation processes. The present work describes the isolation, purification and biochemical characterization of a liver NADPH-dependent cytochrome P450 reductase (CPR) from the marine fish *Liza klunzingeri* (*Lk*CPR). Experimental results revealed that *Lk*CPR is a monomer of approximately 75 kDa that is active in a wide range of pH values (6–9) and temperatures (40–60 °C), showing the highest catalytic activity at pH 8 and 50 °C. The activation energy of the enzyme reaction was 16.3 kcal mol^−1^ K^−1^. The *K_M_* values for cytochrome C and NADPH were 8.83 μM and 7.26 μM, and the *k_cat_* values were 206.79 s^−1^ and 202.93 s^−1^, respectively. *Lk*CPR displayed a specific activity versus cytochrome C of 402.07 µmol min^−1^ mg^1^, the highest activity value described for a CPR up to date (3.2–4.7 times higher than the most active reported CPRs) and showed the highest thermostability described for a CPR. Taking into account all these remarkable catalytic features, *Lk*CPR offers great potential to be used as a suitable biocatalyst.

## 1. Introduction

Nowadays, an alarming increase of seawater pollution (aromatic hydrocarbons, nitrogenous compounds, and heavy metals, among others) is observed. Because chemical degradation of these compounds may produce negative effects on marine living organisms [1], the use of whole cells, enzymes or immobilized biocatalysts to eliminate these toxic compounds (so called bioremediation) is gaining ground. Bioremediation is an effective and eco-friendly solution for these environmental problems [2,3,4,5].

To fulfil the safety requirements of current environmental legislation, the development of new environmentally friendly and cost-effective strategies is needed [6,7].

In this sense, the enzymatic degradation of marine environmental pollutants has come up as a promising alternative that offers high catalytic efficiency and specificity [8,9,10,11]. 

Micro-organisms and enzymes from contaminated marine environments may be of particular interest, as they can function in a wide range of pH and temperature values, in the presence of metal ions, and even at high concentrations of organic solvents [12,13,14].

Cytochrome P450 enzymes (also named CYP450 or CYP) are a large family of hemoproteins that perform different type of oxidation reactions in presence of molecular oxygen [15,16]. P450 systems involve the transfer of one atom of molecular O_2_ to a selected substrate, and then reduce the other atom into water (1).

(1) $$\mathrm{RH}\;+\;O_{2}\;+\;\mathrm{NAD}\;\left(P\right)\;H\;+\;H^{+}\overset{}{\to }\;\mathrm{ROH}\;+\;H_{2}O\;+\;\mathrm{NAD}\;{\left(P\right)}^{+}$$

Due to their capability to oxidize diverse potentially hazardous and toxic substances that can be present in sea water [17,18,19] cytochrome P450 enzyme systems are of particular importance in bioremediation [20,21]. In this sense, great efforts have been applied to discover more adequate CYPs, either searching in nature or modifying already known enzymes [22,23,24]. NADPH-dependent cytochrome P450 reductase (EC 1.6.2.4, CPR) is a key enzyme in P450 systems. CPR firstly acts as an acceptor of two electrons from NADPH or NADH, and subsequently transfers them to cytochrome P450s during catalysis [25]. This paper shows, for the first time to the best of our knowledge, the purification and preliminary characterization of CPR from *Liza klunzingeri* (*Lk*CPR) liver. We also studied the effect of pH and temperature on enzyme activity and stability. Thermodynamic parameters were also determined. Finally, steady-state kinetic studies were performed to determine *K_M_* and *k_cat_*.

## 2. Results and Discussion

### 2.1. Isolation and Purification of the Enzyme

*Lk*CPR was purified by four purification steps (see methods). Table 1 shows the main results. After the first purification step (ammonium sulfate precipitation), an activity of around 12 IU was obtained (specific activity, 12.16 IU/mg) with a purification factor of 1.39. The first anion exchange chromatography (DEAE-Sepharose I) gave a total activity of 10663 IU (specific activity, 30.01 IU/mg), and a purification factor of 3.43. The second anion exchange chromatography (DEAE-Sepharose II) yielded a total activity of 8434 IU (specific activity, 143.70 IU/mg) being a purification factor of 16.51. Finally, the last purification step permitted to recover a total activity of 6579 IU, with a specific activity of 402.07 IU/mg and a purification factor of 46.20 (Table 1). 

Remarkably, *Lk*CPR exhibited a specific activity much higher than the CPRs from other marine organisms; e.g. 8 times higher than *Sc*CPR (60 IU/mg) [26], 9.1 times higher than *Ls*CPR (52.6 IU/mg) [27], 12.6 times higher than CPR from *Rainbow trout* (*Rt*CPR, 38 IU/mg) [28] and 20.6 times higher than CPR from *Sparus aurata* (*Sa*CPR, 23.3 IU/mg) [28]. More interestingly, to our understanding, *Lk*CPR is the most active reported CPR, showing an enzymatic activity that is 3.2- and 4.7-fold higher than the CPRs from Porcine Polymorphonuclear Leukocytes (152 IU/mg) [29], *Saccharomyces cerevisiae* (150 IU/mg) [30] and *Rattus norvegicus* (102 IU/mg) [31], the ones previously described as the most active CPRs. We are analyzing the structural causes for this interesting result (work in progress). 

**Figure 1:**
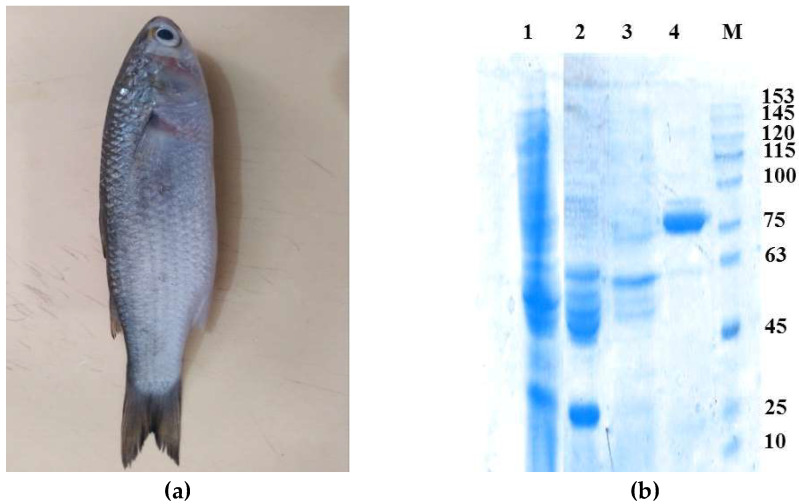
*Liza klunzingeri* fish samples collected from the northern coasts of the Persian Gulf, in Bandar Abbas and after purification of the novel NADPH-dependent cytochrome P450 reductase (for more details see Table 1), the enzyme was analyzed on SDS-PAGE. *Liza klunzingeri* fish (**a**), analysis of 75 kDa novel NADPH-dependent cytochrome P450 reductase on Coomasie brilliant blue -stained SDS-PAGE. SDS-PAGE analysis of samples obtained by the different purification steps. Proteins were detected by Coomassie brilliant blue. Lane 1, crude extract; lane 2, DEAE-Sepharose I pool; lane 3, DEAE-Sepharose II pool; lane 4, 2′,5′-ADP Sepharose 4B pool. Lane M is the molecular mass marker (**b**).

SDS-PAGE gel of the purified enzyme showed a single band around 75 kDa (Figure 1). This molecular weight is similar to that of CPRs from the fishes *Liza saliens* (*Ls*CPR, Mw 77 kDa [27]) and *Alburnus tarichi* (Lake Van fish, *At*CPR, Mw 70 kDa [32], but is lower than the one of the CPR monomer from the marine fish *Stenotomus chrysops* (*Sc*CPR) (Mw 82.6 kDa) [26]. 

**Figure 2:**
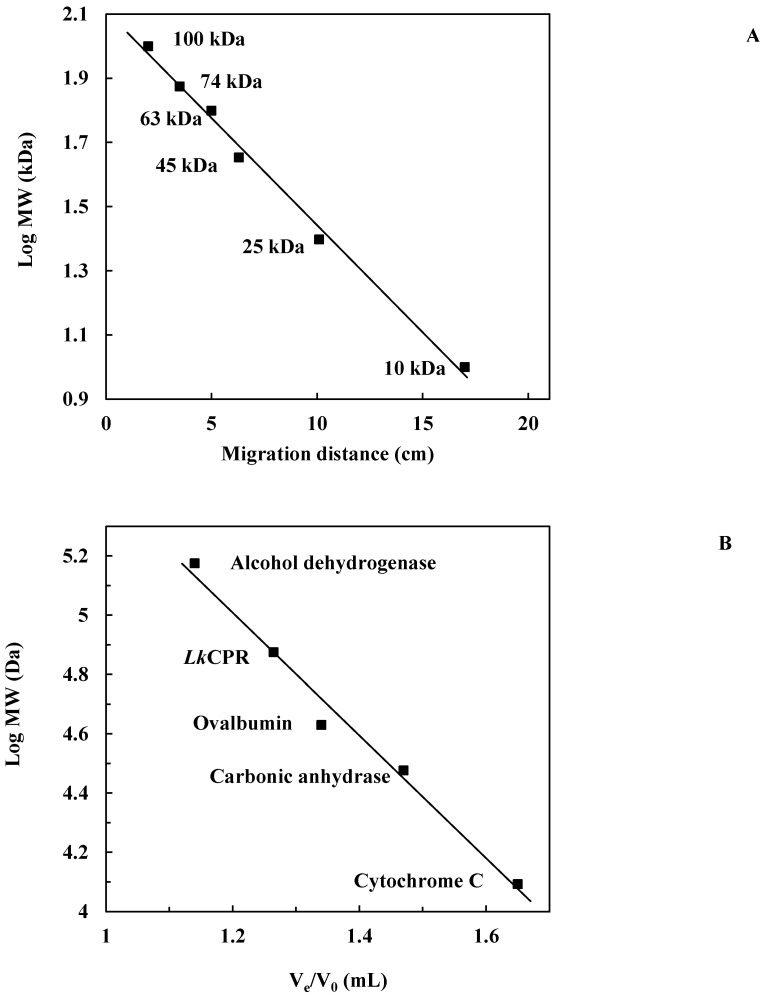
Molecular weight determination of purified *Lk*CPR. Experiments were carried out as described in Methods (**A**). Calibration curve for determination of *Lk*CPR molecular weight by gel filtration chromatography (**B**).

The native molecular weight of *Lk*CPR was found to be approximately 75 kDa, determined by Sephacryl S-200 gel filtration chromatography using an elution calibration curve of standard proteins (Figure 2A). This molecular weight was estimated from comparison of the electrophoretic mobility of *Lk*CPR with the mobilities of marker proteins (Figure 2B). These results reveal that *Lk*CPR is a monomer in solution, similar to yeast cytochrome C reductase (a monomer of 70.0 kDa) [30], the only reported monomeric CPR. However, both CPRs display a different oligomeric state than other reported CPRs (e.g. dimeric CPR from *Trypanosoma cruzi*) [33].

### 2.2. Effect of pH on LkCPR Activity and Stability

**Figure 3:**
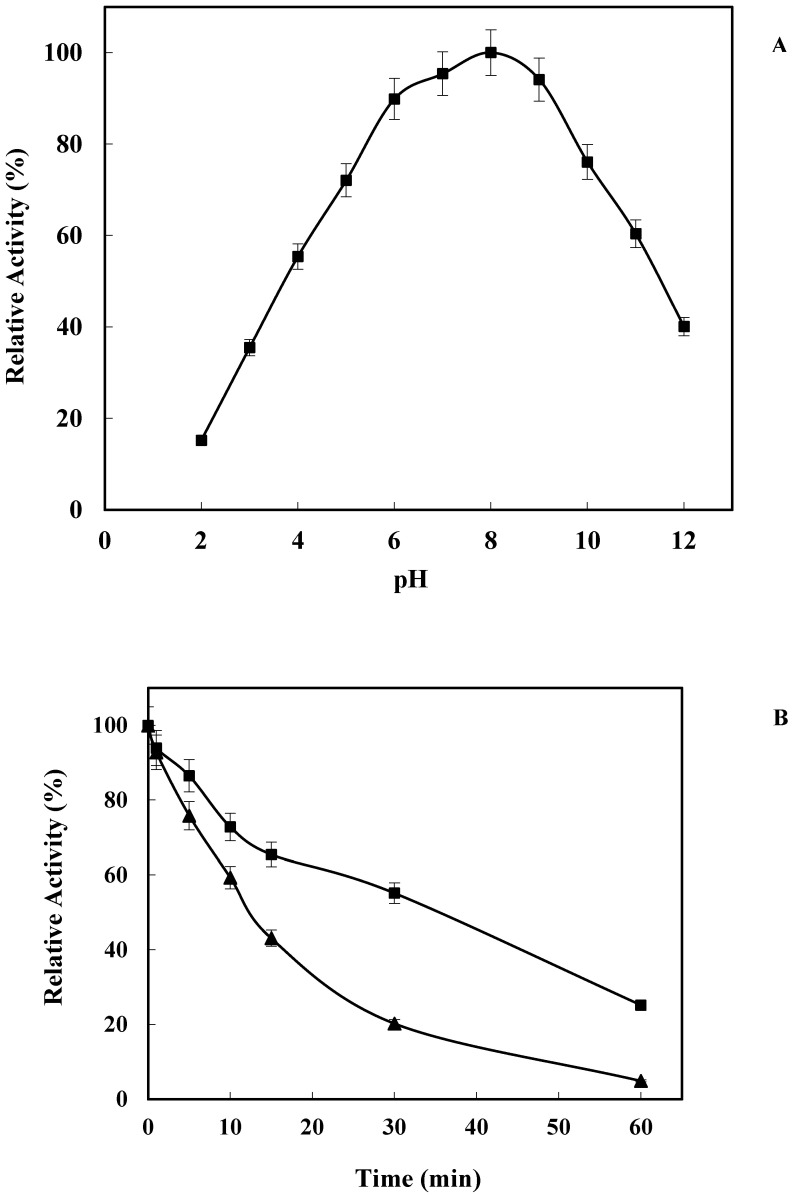
Effect of pH on *Lk*CPR activity. 100% relative activity refers to the percentage of the initial reaction rate obtained by the enzyme at the pH value where maximum activity was detected. The temperature was 25 °C. Experiments were performed as described in Methods (**A**). Inactivation course of *Lk*CPR at pH 3.0 (filled triangles) and 12.0 (filled squares) in mixed buffers at 25 °C. Experiments were performed as described in Methods (**B**).

The enzymatic activity of NADPH-dependent cytochrome P450 reductase remained almost unaltered in a broad pH range (from 5 to 10 the relative activity was >70%), with a maximum value at pH 8 (Figure 3A). These results are similar to results published using other marine fish CPRs, such as *Ls*CPR (90% relative activity in the pH range 7–8) [27] or *At*CPR (82% relative activity in the pH range 7–8) [32], or the CPR from green microalga *Botryococcus braunii* (>90% relative activity in the pH range 7–8) [34]. In addition, *Lk*CPR also displayed around 40% of relative activity at pH 2 and pH 12, values much higher than those reported for other CPR enzymes at these pH values. This activity at extreme pH values may have interest in bioremediation of contaminated sea areas, where the pH values may become extreme under such harsh conditions.

Stability was also studied at these extreme pH values. Figure 3B shows a drastic loss of enzyme activity when the enzyme was incubated 15 min at pH 3 (<50% retained activity). Higher stability was found when the enzyme was incubated at pH 12 (60% retained activity after 30 min). 

### 2.3. Effect of Temperature on LkCPR Activity and Stability

**Figure 4:**
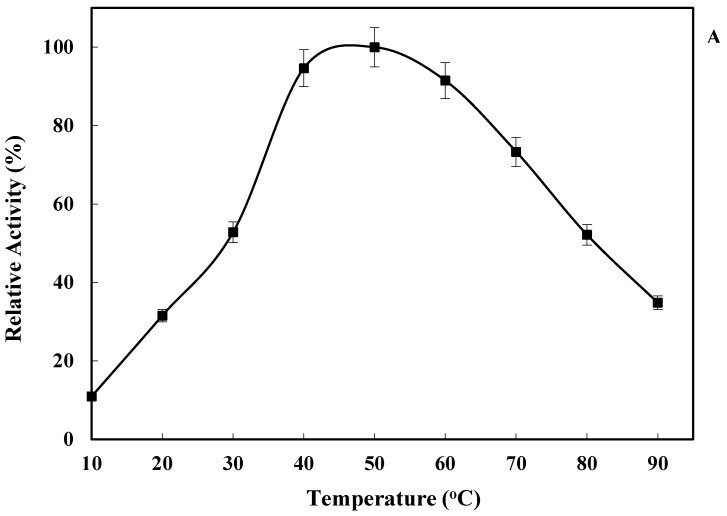

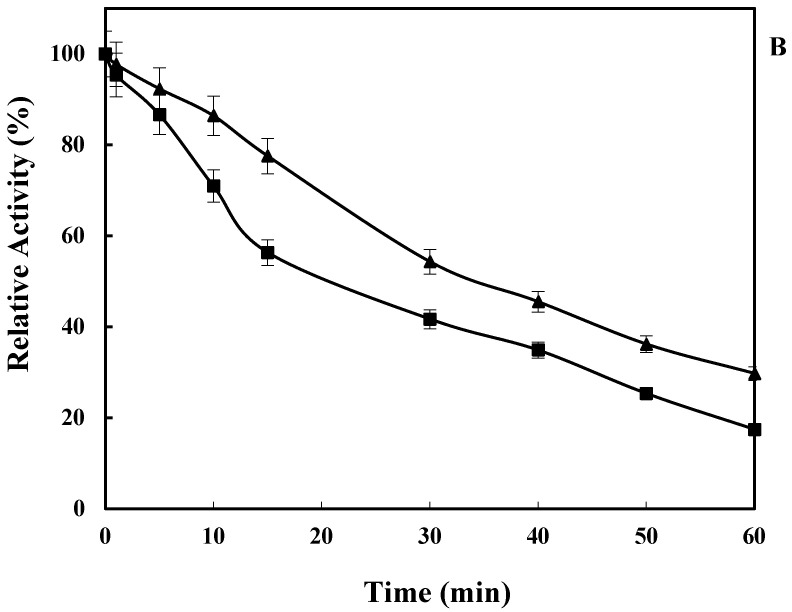
Effect of temperature on *Lk*CPR activity at pH 7.5. The activity at optimal temperature was taken as 100. Other specifications may be found in Methods (**A**). Inactivation course at 80 °C (filled triangles) and 90 °C (filled squares) at pH 7.5 of NADPH cytochrome P450 reductase. The activity of the same enzyme solution, kept on ice, was used as the control (100%). Experiments were performed as described in Methods (**B**).

As shown in Figure 4A, *Lk*CPR exhibited high activity (<70% relative activity) in a temperature range from 40 °C to 70 °C, with a maximum value at 50 °C. Moreover, the enzyme was also quite active even at 90 °C (>30% relative activity). *Lk*CPR showed an optimum temperature similar to that of *At*CPR [32], and higher than the values reported for CPR from the green microalga *Botryococcus braunii* (41 °C) [34]. 

Figure 4B shows that *Lk*CPR showed an unusually high thermal stability even at 80 °C, conditions where the enzyme retained about 60% of the initial activity after 30 min. Furthermore, *Lk*CPR kept 70% of the initial activity after 10 min of incubation at 90 °C. That way, *Lk*CPR exhibits a much higher thermal stability than other purified NADPH- dependent cytochrome P450 reductases, such as those from *Bacillus megaterium* (only 50% activity was retained after 10 min at 54 °C) [35], *Capsicum annuum* (totally inactivated after 10 min at 70 °C) [36], *Rattus norvegicus* (totally inactivated after 2 min at 60 °C) [37]. In fact, we have not found reports on CPR with a higher stability than this new *Lk*CPR.

### 2.4. Steady-State Kinetics

The calculated *K_M_* values for cytochrome C and NADPH were 8.83 μM and 7.26 μM, respectively (Table 2). The *K_M_* values for cytochrome C exhibited by *Lk*CPR are lower than those reported for other marine fish CPRs, such as *At*CPR (12.82 µM for cytochrome C, and 5.20 µM for NADPH) [32] or *Sc*CPR (24 µM for cytochrome C, and 14 µM for NADPH) [26], or the algae *Bb*CPR (11.7 µM for cytochrome C, and 9.4 µM for NADPH) [34]. *k_cat_* values were 202.93 s^−1^ and 206.79 s^−1^ calculated using for cytochrome C and NADPH respectively (calculated *V_max_* values were 25.96 µM min^−1^ (changing cytochrome C concentration) and 26.73 µM min ^−1^ (changing [NADPH] concentration)). This coincidence of *V_max_* using saturating concentrations of each of the substrates confirms the reliability of the results.

According to the Arrhenius plot in the range 20–50 °C (data not shown), the activation energy (*E_a_*) of the enzyme in the reaction was determined to be 16.33 kcal mol^−1^ K^−1^. The influence of temperature on the reaction rate does not provide too much information about the reaction mechanism of the enzyme. However, they can indicate alterations of the enzyme conformation and catalytic performance [7]. In Table 3, the values of the activation enthalpy (Δ*H^#^*), the activation free energy (Δ*G^#^*), and the activation entropy (Δ*S^#^*) are also shown. The low enthalpy value of cytochrome P450 reductase from *Liza klunzingeri* suggests a very efficient production of the transition state. Moreover, the low *∆G^#^* value points to a great tendency of this transition state to give the corresponding products. The change in Gibbs free energy *(∆G^#^*) is a good indicator of the feasibility of chemical reaction, i.e., the transformation of ES complex into the corresponding products [7]. The great affinity of the enzyme towards its substrates was confirmed by the activation free energy of substrate binding (∆*G^#^_E–S_*) and the free energy of the formation of the activation complex (∆*G^#^_E–T_*) (1.36 and −2 kcal mol^−1^, respectively). 

## 3. Materials and Methods

### 3.1. Materials

All reagents were purchased from Merck (Darmstadt, Germany).

### 3.2. Collecting Samples and Extraction of Protein Extracts from Liver Microsomal Cells

The specimens used in this study (15 samples of *Liza klunzingeri* fish, with an approximate weight of 300 g per sample) were captured in September 2017 from the northern coasts of the Persian Gulf, in Bandar Abbas. The collected fish were washed with marine water and packed in clean plastic bags and conserved in ice, transported to the laboratory, and frozen at −80 °C. They were washed with distilled water and the liver samples were extracted by surgery. The liver samples were washed with 20 mM KCl/2 mM EDTA (pH 7.5). All subsequent steps were carried out at temperatures between 0 °C and 4 °C. 

The liver samples were homogenized in 50 mM sodium phosphate buffer, pH 7.5, containing 0.5 mM ε-ACA, 0.5 mM PMSF and 5 mM EDTA. This solution was centrifuged at 20,000 rpm for 30 min at 4 °C and the supernatant was collected. This was centrifuged again at 25,000 rpm for 60 min at 4 °C. The final precipitated microsomes were resuspended in a minimum amount of 20 mM KCl/2 mM EDTA (pH 7.5). After a second centrifugation the microsomal precipitate was resuspended in a minimum aqueous solution of 10% (*v/v*) glycerol/2 mM EDTA. This suspension was diluted in 50 mM sodium phosphate (pH 7.5), containing 20 μM BHT, 0.5 mM PMSF, 5 mM EDTA, 0.1 mM DTT, 0.5 mM ε-ACA, 0.5% (*w/v*) Na-cholate, 0.5% (*w/v*) Emulgen 913 and 25% (*v/v*) glycerol. The suspension containing microsomes was incubated in Emulgen 911 and centrifuged at 4 °C for 60 min at 25,000 rpm. The yellow supernatant was separated from the sediment and kept at 4 °C.

### 3.3. Isolation and Purification of LkCPR

#### 3.3.1. Ammonium Sulfate Precipitation

The yellow solution obtained above was poured into a small beaker, and the beaker was placed in an ice container and submitted to magnetic stirring. Then, solid ammonium sulfate was slowly added at 4 °C up to 20% saturation. The suspension was centrifuged at 12,000 rpm for 20 min and solid ammonium sulfate was added to the above supernatant to reach 85% ammonium sulfate saturation and stirred at 4 °C. After a new centrifugation, the precipitate was resuspended in 50 mM Tris-HCl buffer at pH 7.5 and dialyzed against the same buffer for 24 h at 4 °C, performing three dialysis buffer changes (one every 8 h).

#### 3.3.2. Purification and Determination of Molecular Weight

The dialyzed protein samples were subjected to ion exchange chromatography using a DEAE−Sepharose fast flow column (Sigma-Aldrich, St. Louis, MO, United States). The column (2.5 × 6.5 cm) was pre-equilibrated with 50 mM sodium phosphate buffer at pH 7.5 (buffer A) and then the sample was applied at a flow rate of 0.5 mL/min. Then, the column with the ionically exchanged proteins was extensively washed with buffer A until no protein was detected in the outlet. With a linear gradient of 0–1 M NaCl added to the buffer A, using 0.5 mL/min flow rate the bound proteins were eluted. The protein elution was followed by UV–vis spectroscopy at 280 nm. The activity of the different samples was assayed using cytochrome C as substrate (standard assay). 

The fractions exhibiting activity were collected, dialyzed against buffer A, concentrated by an Amicon filter (Millipore Cork, Biochrom GmbH, Danvers, MA, United States), and subjected to re-chromatography in the same column as described above. The fractions exhibiting enzymatic activity were selected and dialyzed versus buffer A, concentrated, and applied to a 2′,5′-ADP Sepharose 4B column, pre-equilibrated with buffer A. The bound proteins were eluted using an NaCl gradient of 0–1 M in buffer A, using a flow rate of 0.5 mL/min. After the elution, fractions with enzymatic activity were collected, dialyzed and concentrated as described above. Finally, purified protein was dialyzed against 50 mM Tris-HCl buffer at pH 7.5 for 24 h and stored at −20 °C prior to use. 

To determine the oligomer state of enzyme in solution, gel filtration chromatography was performed. The purified enzyme was applied to a Sephacryl S-200 column, pre-equilibrated with 50 mM potassium phosphate buffer (pH 7.5), at 0.5 mL/min flow rate. The column was calibrated using the following four standard proteins: alcohol dehydrogenase (150 kDa), ovalbumin (42.7 kDa), carbonic anhydrase (30 kDa), and cytochrome C (12.4 kDa). The plot Ve/Vo versus log molecular weight for these proteins was used to determine the molecular weight; in this plot, Ve and Vo are the volume of elution for each protein and the volume of void determined by blue dextran, respectively. 

SDS-PAGE was performed using a 15% polyacrylamide slab gel equilibrated with 25 mM Tris-HCl buffer at pH 8.6 containing 0.1 (*w/v*) % SDS. Protein concentration was determined following Bradford’s method using bovine serum albumin (BSA) as standard [38]. 

### 3.4. Enzymatic Activity Assay

The activity of *Lk*CPR was determined by monitoring the increase of absorbance at 550 nm, which is related to cytochrome C reduction [39]. In this assay, NADPH- dependent cytochrome P450 reductase (CPR) takes electrons from NADPH and transfers them to cytochrome C. The reaction was performed using 0.36 mg of pure protein solution in 1 mL of 0.3 M potassium phosphate buffer (pH 7.5) containing 50 mM of cytochrome C at room temperature [34]. The extinction coefficient of ferrous to ferric cytochrome is described to be 19.6 mM^−1^ cm^−1^ [40,41]. One activity unit (IU) was defined as the amount of enzyme (in mg) which catalyzes the reduction of 1 µmol of cytochrome C per min under the above indicated assay conditions.

### 3.5. Biochemical Characterization of LkCPR 

#### 3.5.1. Effect of Temperature on Enzyme Activity and Stability

The influence of temperature on the enzymatic activity was determined using the standard assay in the 10–90 °C temperature range, at pH 7.5. Moreover, the thermal stability of *Lk*CPR was determined by incubating the purified enzyme in 50 mM phosphate buffer, pH 7.5, at 80 °C and 90 °C for a period of 60 min. During incubation, samples were taken at different time periods, and after cooling, the activity was measured using the standard assay.

#### 3.5.2. Effect of pH on Enzyme Activity and Stability

The influence of pH on *Lk*CPR activity was analyzed employing the standard assay determining the enzyme activity from pH 2 to 12, mixing equal amounts of 50 mM sodium acetate, 50 mM sodium phosphate, 50 mM Tris-HCl and 50 mM sodium carbonate to counteract the buffer nature effect on enzyme activity. In stability studies, *Lk*CPR was incubated at room temperature at pH 3 and pH 12 for 60 min, and the activity was measured using the standard assay.

#### 3.5.3. Determination of Kinetic Parameters

The *K_M_, k_cat_*, and *V_max_* were calculated under standard conditions by determining the enzyme activity at different concentrations of one of the substrates (10–100 µM cytochrome C or NADPH), while using saturation concentration for the other substrate (50 µM). Apparent *K_M_, k_cat_*, and *V_max_* were calculated assuming Michaelis–Menten kinetics using the GraphPad Prism 5 software [42].

#### 3.5.4. Calculation of Thermodynamic Parameters

The constants of inactivation (*k_inact_*) and enzyme activity (*k_cat_*) were utilized to calculate the activation energy following Arrhenius equation [43].

(2) $$k=Ae^{\frac{-E_{a}}{RT}}$$

where *A* is a pre-exponential factor related to the molecular collision frequency and steric effects, *k* (s^−1^) is the rate constant at a specific temperature *T* (K), *E_a_* is the activation energy of the reaction and R is the gas constant (8.314 J mol^−1^ K^−1^). Representing ln *k* as a function of 1/T produces a straight line with *−E_a_/R* as slope. The thermodynamic parameters of activation were calculated as previously stated [44]:

(3) $$\Delta G^{\#}=-RT\ln(\frac{k_{B}T}{h})-RT\ln k_{cat}$$

(4) $$\Delta H^{\#}=E_{a}-RT$$

(5) $$\Delta S^{\#}=\frac{\left(\Delta H^{\#}-\Delta G^{\#}\right)}{T}$$

(6) $$\Delta G^{\#}{}_{E-S}=-RT\ln\frac{1}{K_{m}}$$

(7) $$\Delta G^{\#}{}_{E-T}=-RT\ln(\frac{k_{cat}}{K_{m}})$$

where *k_cat_*(s^−1^) is the rate constant at *T* (K), *h* is Planck’s constant (6.6256×10^−34^ J s) and *k_B_* is the Boltzmann constant (1.3805×10^−23^ J K^−1^).

## 4. Conclusions

Herein we report for the first time, the purification and partial characterization of an NADPH-dependent cytochrome P450 reductase from *Liza klunzingeri* liver (*Lk*CPR). Its biochemical characterization revealed that *Lk*CPR is a monomer with high activity in a broad range of pH (from 5 to pH 10) and temperature (from 40 to 70 °C) range, with pH 8 and 50 °C being the optimal conditions. *Lk*CPR displayed lower *K_M_* values as well as a higher catalytic performance than other marine CPRs. Furthermore, *Lk*CPR showed an unusually high stability at high temperatures and extreme pH values, the highest ones among the reported CPRs. Additionally, the enzyme exhibits the highest specific activity reported to date for a CPR. 

Interestingly, some of the marine CPRs mentioned (namely, *Sc*CPR [26] and *Ls*CPR [27]) have been able to metabolize different substrates when reconstituted with CYPs. In particular, the system comprising both cytochrome P-450E and *Sc*CPR was active on aromatic hydrocarbons [26]. The fact that *Lk*CPR displays improved activity and higher temperature and pH stability when compared to its marine counterparts, leads us to believe that this enzyme could act as an optimal redox partner in a CYP-containing system.

In a more practical example, cytochrome CYP71B1 and NADPH cytochrome P450 reductase were immobilized through colloidal liquid aphrons (CLAs) [45]. Then, the obtained biocatalyst was successfully employed for the degradation of chlortoluron, an organic compound of phenylurea herbicides. Compared with the free enzyme, the immobilized biocatalyst displayed higher enzymatic and retained activity. This type of immobilized derivative arises as an interesting strategy for the development of robust catalysts for bioremediation. However, the harsh conditions often required for the immobilization process (alkaline conditions and long reaction times) could adversely affect the enzyme. In this sense, *Lk*CPR could be a suitable candidate for the development of immobilized biocatalysts, since it exhibits remarkable stability and activity under extreme conditions.

To date, in the light of all the presented data, cytochrome P450 reductase from *Liza klunzingeri* arises as a suitable biocatalyst for the removal of marine pollutants.

## Figures and Tables

**Table 1:** Purification procedures of NADPH-cytochrome P450 enzyme from *Liza klunzingeri* liver.

Purification Steps	Total Protein (mg)	Total Activity (IU)	Specific Activity (IU/mg)	Purification Fold	Yield (%)
Cell extract	1683 ± 156	14,732 ± 2160	8.70 ± 0.75	1 ± 0.00	100 ± 0.00
(NH_4_)_2_SO_4_	1009 ± 128	12,316 ± 1983	12.16 ± 0.59	1.39 ± 0.05	83 ± 1.52
DEAE-Sepharose I	352 ± 43	10,663 ± 2351	30.01 ± 3.50	3.43 ± 0.12	71 ± 5.85
DEAE-Sepharose II	58 ± 13	8434 ± 2500	143.70 ± 11.62	16.51 ± 0.46	56 ± 9.07
2′,5′-ADP Sepharose 4B	16 ± 6	6579 ± 2968	402.07 ± 35.10	46.20 ± 2.08	42 ± 14.74

**Table 2:** The kinetic parameters for cytochrome C and NADH catalyzed by *Liza klunzingeri* liver NADPHcytochrome P450 reductase.

Parameters	Substrate	
	Cytochrome C	NADPH
*K_m_* (µM)	8.83 ± 0.15	7.26 ± 0.20
*V_max_* (µM·min^−1^)	25.96 ± 0.30	26.73 ± 0.35
*k_cat_* (s^−1^)	202.93 ± 0.53	206.79 ± 33
*k_cat_/K_m_* (s^−1^ µM^−1^)	22.05 ± 0.59	27.57 ± 0.84

**Table 3:** Thermodynamic parameters for cytochrome C catalyzed by *Lk*CPR.

*E_a_* (kcal mol^−1^)	Δ*H^#^* (kcal mol^−1^)	Δ*G^#^* (kcal mol^−1^)	Δ*S^#^* (cal molK^−1^)	Δ*G^#^_E-S_* (kcal mol^−1^)	Δ*G^#^_E-T_* (kcal mol^−1^)
16.33 ± 0.20	15.73 ± 0.20	14.40 ± 0.35	4.43 ± 0.51	1.36 ± 0.15	−2 ± 0.14

Conditions: 50 mM phosphate buffer, pH 7.5, using 50 µM cytochrome C as substrate. Each number is the average of at least three independent experiments.

## Data Availability

All data were included in the manuscript.
